# Preventive effect of bentonite against pyoderma via direct binding capability of staphylococci

**DOI:** 10.1371/journal.pone.0341148

**Published:** 2026-01-23

**Authors:** Mao Kaneki, Chiharu Ohira, Miyu Takahashi, Tomoki Fukuyama

**Affiliations:** 1 Laboratory of Veterinary Pharmacology, School of Veterinary Medicine, Azabu University, Sagamihara-shi, Kanagawa, Japan; 2 Center for Human and Animal Symbiosis Science, Azabu University, Sagamihara-shi, Kanagawa, Japan; University of Central Florida, UNITED STATES OF AMERICA

## Abstract

Bentonite, a naturally occurring clay mineral, is widely used for detoxifying mycotoxins and chemical contaminants, yet its potential interactions with microorganisms remain poorly characterized. Staphylococci, including *Staphylococcus aureus* in humans and *Staphylococcus pseudintermedius* in dogs, are major contributors to impetigo or pyoderma and other skin diseases, with rising antibiotic resistance underscoring the need for novel preventive strategies. This study investigated whether sodium bentonite directly modulates staphylococcal growth and associated inflammatory responses *in vitro* and *in vivo*. In bacterial culture assays, sodium bentonite showed a strong binding affinity for staphylococci, leading to significant reductions in viable colony counts. Pre-treatment of *S. aureus* and *S. pseudintermedius* with bentonite attenuated keratinocyte injury and markedly suppressed pro-inflammatory cytokine secretion, including IL-6, IL-8, and CCL2. In a murine pyoderma model, bentonite pre-treatment of *S. pseudintermedius* prevented lesion development, reduced bacterial burden, and downregulated cutaneous expression of TNFα, IL-1β, and IL-13, findings that were corroborated by histological improvements. Importantly, these effects extended to methicillin-resistant strains, highlighting a potential application against drug-resistant staphylococcal infections. By contrast, direct topical administration of bentonite gel in a murine atopic dermatitis model produced only modest benefits: ear swelling was reduced, but transepidermal water loss and clinical scores remained unchanged, suggesting limited therapeutic value in established chronic inflammatory conditions. Overall, sodium bentonite does not exhibit intrinsic bactericidal or anti-inflammatory properties but exerts protective effects through direct bacterial binding and subsequent inhibition of staphylococcus-induced cytotoxicity and inflammation. These findings identify sodium bentonite as a safe, non-antibiotic preventive option for staphylococci-associated skin diseases, particularly pyoderma, in both humans and companion animals. Future studies should further explore formulation strategies and delivery methods to optimize its clinical utility.

## Introduction

Staphylococci are major contributors to inflammatory skin diseases such as atopic dermatitis (AD), psoriasis, and pyoderma [1–3]. *Staphylococcus aureus* is particularly important in humans, where it exacerbates AD by disrupting the skin barrier and triggering cytokine production by immune cells. Recent findings further highlight its pathogenic role, showing that dermal colonization by *S. aureus* can drive Th17-mediated immune responses that intensify itching and pain [4]. In veterinary medicine, pyoderma is one of the most prevalent canine skin disorders worldwide [5]. It is most frequently caused by *Staphylococcus pseudintermedius* (*S. pseudintermedius*), a coagulase-positive bacterium that can also exhibit methicillin resistance [6,7]. Other staphylococci, such as *S. aureus* and *S. schleiferi*, may also be involved. Dogs with underlying conditions, particularly AD, are predisposed to recurrent pyoderma, with allergic animals showing significantly higher staphylococcal colonization compared with healthy counterparts [8–10].

Treating staphylococcal skin infections remains challenging because of rising antibiotic resistance. Multidrug-resistant *S. pseudintermedius* and methicillin-resistant *S. aureus* (MRSA) not only prolong treatment and recovery times but also require stronger antibiotics, which may carry adverse effects and further fuel resistance. This has created a pressing demand for effective, antibiotic-independent alternatives to manage staphylococcal infections in both humans and animals [5]. Several antiseptic approaches have been investigated as alternatives, including ozone water, which has demonstrated bactericidal effects against both *S. aureus* and methicillin-resistant *S. pseudintermedius* in experimental models. Although promising, ozone water presents limitations such as instability, challenges in maintaining effective concentrations, and potential adverse effects on skin tissues. Thus, the search continues for safe, stable, and practical interventions [11].

Bentonite, a naturally occurring clay mineral, offers potential in this regard. It has long been used as a feed additive without restriction under European Commission regulations and is known for its strong adsorptive properties [12]. Previous research has demonstrated its effectiveness in detoxifying mycotoxins such as aflatoxin B1 and ochratoxin A, as well as chemical pollutants like benzene [13,14]. Despite these properties, the interactions between bentonite and microorganisms, including staphylococci, remain largely unexplored. Given its binding capacity, bentonite may serve as a non-antibiotic approach to preventing staphylococcal infection and the associated inflammatory responses. By physically adsorbing bacteria, bentonite could reduce colonization, mitigate keratinocyte injury, and suppress cytokine production that drives inflammation. Importantly, such an approach may be relevant to both human and veterinary medicine, particularly in the context of pyoderma, where recurrent infections and resistant strains complicate management.

The present study therefore aimed to investigate the direct effects of sodium bentonite on staphylococci in vitro and in vivo. Specifically, we evaluated whether bentonite could inhibit bacterial growth, prevent cytotoxicity, and reduce cytokine secretion in human keratinocytes. In parallel, we examined its efficacy in murine models of staphylococcus-induced pyoderma and atopic dermatitis to assess its preventive and therapeutic potential. Through this work, we sought to establish whether sodium bentonite could serve as a safe, effective, and antibiotic-independent preventive strategy against staphylococci-associated skin diseases in humans and companion animals.

## Materials and methods

### Bentonite

Pulverized sodium bentonite extracted from the Hosogoe mine (Niigata, Japan) was used in this study (Kunimine Industries Co., Ltd., Tokyo, Japan).

### Preparation of staphylococcal strains

*S. aureus* strain SA27, isolated from a healthy individual, has been described previously, and *S. pseudintermedius* strain D1, a methicillin-resistant bacterium, was isolated from dogs with pyoderma [15,16]. All strains of staphylococci were aerobically cultured in a liquid Luria-Bertani (LB) broth (pH 7.0 ± 0.2, Kanto Chemical Co., Inc., Tokyo, Japan) at 37°C. They were cultured at mid-log phase, as indicated by an optical density (OD, 600 nm) of 0.3–0.35 (1 × 10⁷–1 × 10⁸ colony-forming units [CFU]/mL).

### Binding property of bentonite with *S. pseudintermedius*

To visualize the binding interaction between bentonite and *S. pseudintermedius* strain D1, bacterial cells were incubated with sodium bentonite for 15 min at room temperature and then placed onto glass coverslips for imaging. Samples were examined by scanning electron microscopy (SEM) using a Miniscope® TM4000Plus III (Hitachi High-Tech Corporation, Tokyo, Japan).

### Inhibitory effects of bentonite on the growth of staphylococcal strains

**Fig 1:**
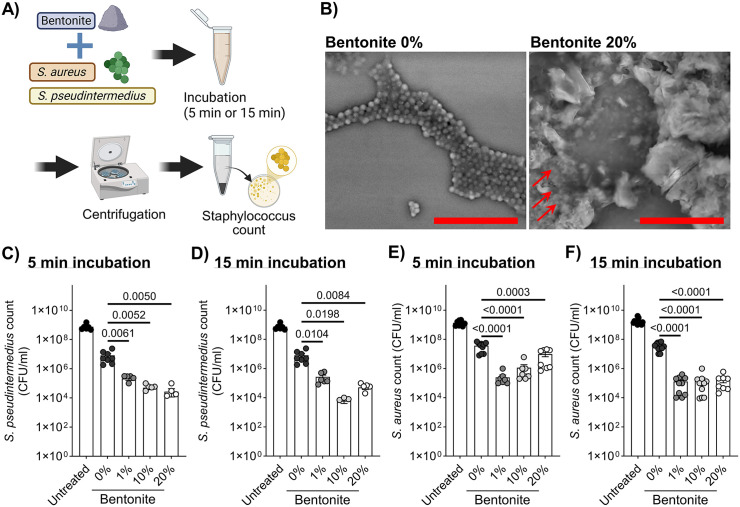
Inhibitory effects of sodium bentonite on staphylococcal viability. **(A)** Schematic of bentonite exposure of staphylococci to evaluate the inhibition of bacterial growth. **(B)** Representative scanning electron microscopy images of *S. pseudintermedius* strain D1 after 15 min exposure to sodium bentonite (Bar = 10 μm). Impact of bentonite pre-treatment for **(C)** 5 min and **(D)** 15 min on the growth of *S. pseudintermedius* strain D1. A similar inhibitory trend was observed when *S. aureus* strain SA27 was pre-treated with several concentrations of bentonite for **(E)** 5 min and **(F)** 15 min. Each result is presented as the mean ± standard error of the mean (SEM). n = 6–16 per group. *p* < 0.05 (Dunnett’s multiple comparisons test) vs. the 0% group. *S. pseudintermedius*, *Staphylococcus pseudintermedius*; *S. aureus*, *Staphylococcus aureus*.

Bacterial suspensions were mixed with different concentrations of bentonite (0, 1, 10, 20%) and were incubated for 5 min or 15 min at room temperature. After centrifugation (12,000 rpm, 5 min; CFM-100; AGC Techno Glass Co., Ltd., Tokyo, Japan), the resulting supernatants were plated on LB agar aerobically at 37°C for 24 h and number of CFU was measured (Fig 1A). Each condition included 6–16 replicates, and all experiments were independently repeated three times.

### Cytotoxicity and inflammatory cytokine release induced by staphylococcal strains in human epidermal keratinocytes

**Fig 2:**
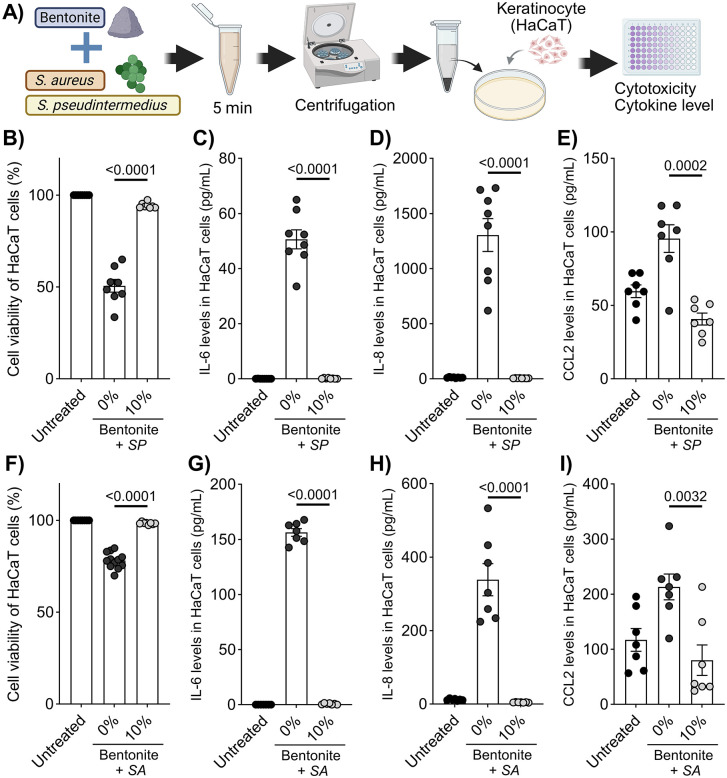
Bentonite pre-treatment reduces staphylococcal cytotoxicity and inflammatory responses in human keratinocytes. **(A)** Schematic of bentonite exposure of staphylococci to evaluate cell viability and cytokine production in human keratinocytes (HaCaT cells). Pre-treatment of *S. pseudintermedius* strain D1 with 10% bentonite significantly inhibited **(B)** *in vitro* cytotoxicity and **(C)** IL-6, **(D)** IL-8, and **(E)** CCL2 secretion by HaCaT cells. Each result is presented as the mean ± SEM. n = 7–8 per group. *p* < 0.05 (unpaired Student’s t test) vs. the 0% group. CCL, C-C motif chemokine ligand; IL, interleukin; SP, *S. pseudintermedius* strain D1; SA, *S. aureus* strain SA27; CFU, colony-forming units.

HaCaT cells (human epidermal keratinocytes) were obtained from Cell Lines Service, GmbH (Eppelheim, Germany) and cultured in Dulbecco’s modified Eagle medium (Fujifilm Wako Pure Chemical Corporation, Osaka, Japan) supplemented with 10% fetal calf serum (Sigma-Aldrich Co. LLC., Tokyo, Japan) and penicillin-streptomycin (Fujifilm Wako Pure Chemical Corporation). Bacterial suspensions were mixed with bentonite (0, 10%) and were incubated for 5 min at room temperature. After centrifugation (12,000 rpm, 5 min), the resulting supernatants were mixed with DMEM (FCS and antibiotic-free) in equal amounts. Then they were added to the HaCaT cells (1 × 10^4^ cells) at 70% confluence in 96-well plates. After 24 h of incubation (37°C and 5% CO_2_), cell viability was monitored based on lactase dehydrogenase release (Cytotoxicity LDH Assay Kit-WST; Dojindo Laboratories, Kumamoto, Japan), and the concentrations of interleukin (IL)-6, IL-8 and C-C motif chemokine ligand (CCL) 2 in the supernatants were quantified using enzyme-linked immunosorbent assays (DuoSet ELISA kit; R&D Systems, Minneapolis, MN, USA) (Fig 2A). Each condition included 7–8 replicates, and all experiments were independently repeated three times.

### Experimental animals

Seven-week-old female BALB/c and NC/Nga mice were provided by Japan SLC, Inc. (Shizuoka, Japan) to develop the pyoderma and AD models, respectively. All mice were housed in controlled conditions with a 12 h light/dark cycle, a temperature of 22 ± 3°C, and humidity at 50 ± 20%. The mice were provided food and water *ad libitum*. All experiments were conducted in accordance with the Animal Care and Use Program of Azabu University (approval no. 220316−47). All methods are reported in accordance with ARRIVE guidelines (https://arriveguidelines.org), relevant guidelines and regulations.

### Murine model of pyoderma

**Fig 3:**
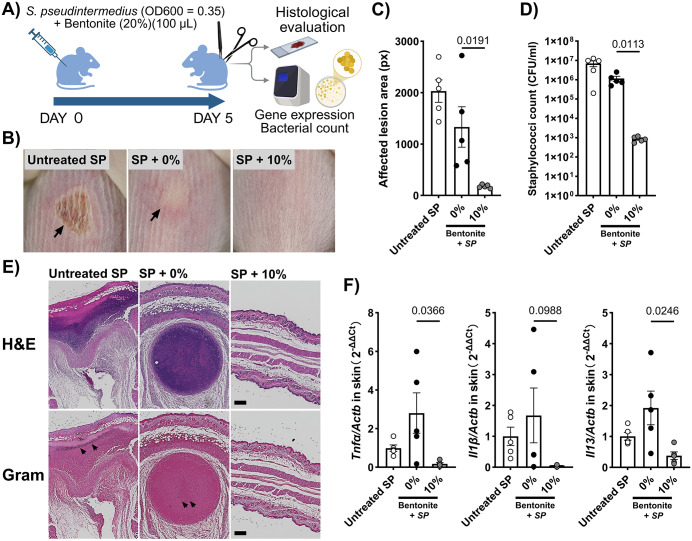
Bentonite pre-treatment suppresses staphylococcal pyoderma development in a mouse model. **(A)** Experimental protocol in a mouse model of pyoderma. Pre-treatment of *S. pseudintermedius* strain D1 with 10% bentonite significantly inhibited the development of pyoderma in mice including the **(B, C)** *S. pseudintermedius*-induced lesion area and **(D)** staphylococcal count. **(E)** Representative histological images of the affected skin in each group. **(F)** Inflammatory gene expression levels in the skin were also significantly reduced by bentonite pre-treatment. Each result is presented as the mean ± SEM. n = 5 per group. p < 0.05 (unpaired Student’s t test) vs. the 0% group.

A murine model of pyoderma was developed through intracutaneous administration (the depilated back area) of *S. pseudintermedius* strain D1 (100 μL) in BALB/c mice, as described previously [11] (Fig 3A). To determine the direct effect of bentonite on *S. pseudintermedius*, an experiment was conducted with separate groups of mice (n = 5/group), with each group receiving no treatment or treatment with 10% (w/v) sodium bentonite prepared as an aqueous suspension. The suspension was freshly prepared before use by dispersing bentonite powder in sterile distilled water under continuous stirring to ensure uniform hydration. Five days after bentonite administration, the size of the affected lesion was recorded using a Dermo camera (DZ-D100; Casio Computer Co., Ltd., Tokyo, Japan). Mice were then humanely euthanized under deep anesthesia with 5% isoflurane, followed by exsanguination, after which back skin samples were collected. A portion of each skin sample was gently washed with sterile phosphate-buffered saline (Thermo Fisher Scientific Inc., Kanagawa, Japan) prior to further analysis. The bacterial suspension was plated on mannitol salt agar (Fujifilm Wako Pure Chemical Corporation), and the bacterial colonies were counted 24 h after aerobic incubation at 37°C. A second portion of the skin sample was fixed in 10% formalin, embedded in paraffin wax, sectioned to a thickness of 5 μm, and stained with either hematoxylin and eosin or Gram stain. Semi-quantitative histopathological evaluation of epidermal hyperplasia, crust, cellular infiltration, necrosis, and abscesses and the examination of the presence of gram-positive bacteria in the epidermis, dermis, and muscle layer were performed in a blinded fashion using the following grading system: 0, within normal limits; 1, mild; 2, moderate; and 3, severe. The total lesion score was used for statistical analysis. The final portion of skin tissue was homogenized, and total RNA was extracted using a NucleoSpin^®^ RNA kit (TaKaRa Bio Inc., Shiga, Japan). Total RNA (500 ng) was reverse transcribed using the PrimeScript™ RT Master Mix (TaKaRa Bio Inc.). The expression levels of β-actin, *TNFα, IL-1β,* and *IL-13* genes were assessed using specific primers (Takara Bio Inc.), PowerUp™ SYBR™ Green Master Mix (Thermo Fisher Scientific Inc.), and a qPCR system (CFX Duet Real-Time PCR System; Bio-Rad Laboratories Inc., Tokyo, Japan). The expression of each gene was normalized to that of β-actin.

### Mouse model of AD

**Fig 4:**
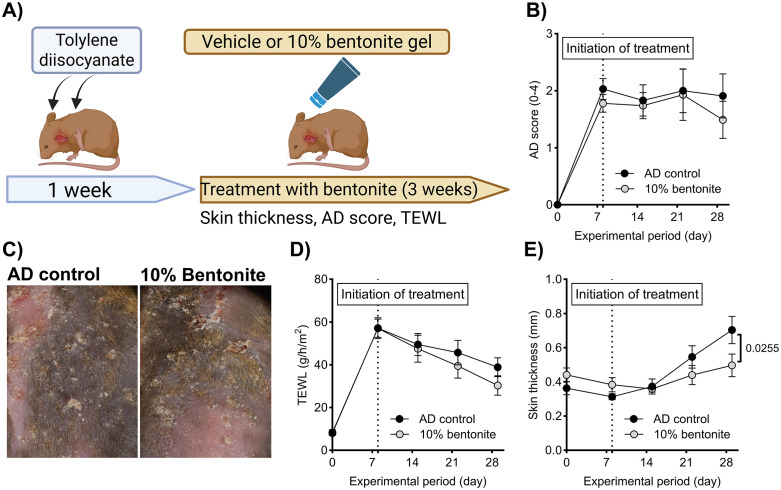
Topical bentonite treatment modestly reduces ear swelling without altering lesion severity in a mouse model of atopic dermatitis. **(A)** The experimental protocol for developing a mouse model of AD and bentonite treatment. Topical treatment with a 10% bentonite gel did not affect **(B, C)** AD lesion formation or **(D)** the cutaneous barrier in a mouse model of AD; however, **(E)** ear thickness was significantly lower in the bentonite-treated group than the vehicle-treated group. Each result is presented as the mean ± SEM. n = 10 per group. *p* < 0.05 (Šídák’s multiple comparisons test) vs. the AD control group. AD, atopic dermatitis; TEWL, transepidermal water loss.

The mouse model of AD was generated using topical sensitization and challenge with 5% tolylene-2,4-diisocyanate (TDI; Fujifilm Wako Pure Chemical Corporation) on the depilated back skin and the ear auricles of NC/Nga mice, as previously described [11] (Fig 4A). In the therapeutic context, the treatment regimen involved daily topical application of a gel containing 10% bentonite (n = 10) or a vehicle gel (n = 10). Sodium bentonite was formulated at 10% (w/w) in caprylic/capric triglycerides, a neutral, dermatologically accepted emollient commonly used for topical delivery. Vehicle-control mice were treated with the identical formulation lacking bentonite. Treatment was initiated on day 0, which was 8 days after the development of AD, when the mean AD score was 1.91. Transepidermal water loss (TEWL), ear skin thickness, and clinical scores were monitored weekly during the experimental period. TEWL was measured using a VAPO SCAN (AS-VT100RS, ASCH JAPAN Co., Ltd, Tokyo, Japan), and a clinical score from 0 to 4 was assigned as follows: no symptoms, 0; mild, 1; moderate, 2; severe, 3; and extreme, 4, as described previously [11]. At the end of the experiment, mice were euthanized under deep anesthesia with 5% isoflurane followed by exsanguination.

### Statistical analyses

Data are expressed as the mean ± standard error of the mean. Analysis of variance followed by Dunnett’s multiple comparisons test, Šídák’s multiple comparisons test, or Student’s t-test were used to evaluate the results. Statistical significance was set at a 5% significance level. Data were analyzed using GraphPad Prism 10 (GraphPad Software, San Diego, CA, USA).

## Results

### Bentonite inhibits staphylococcal growth in vitro

SEM analysis revealed a clear binding interaction between sodium bentonite and *S. pseudintermedius*, characterized by prominent bacterial clustering around bentonite particles and a noticeable reduction in extracellular matrix compared with the control group (Fig 1B). Incubation of *S. pseudintermedius* strain D1 with sodium bentonite resulted in a concentration-dependent reduction in bacterial viability. Significant inhibition was observed at 1, 10, and 20% bentonite after both 5 and 15 min of exposure (p < 0.05 vs. untreated control). A similar pattern was detected for *S. aureus* strain SA27, with 15 min of pre-incubation producing marked growth suppression across all tested concentrations. These findings indicate that sodium bentonite rapidly binds and sequesters staphylococci, thereby limiting bacterial proliferation (Fig 1C–1F).

### Bentonite pre-treatment reduces keratinocyte cytotoxicity and inflammatory cytokine release

To evaluate whether bacterial neutralization by bentonite alters host responses, HaCaT keratinocytes were exposed to staphylococcal suspensions pretreated with 10% bentonite. Both *S. pseudintermedius*- and *S. aureus*-induced cytotoxicity were significantly reduced compared with untreated bacteria, as reflected by lower LDH release (p < 0.01). In addition, bentonite pre-treatment markedly suppressed the secretion of IL-6, IL-8, and CCL2, key mediators of skin inflammation, in keratinocyte cultures (p < 0.05 for all comparisons). These results suggest that bentonite-mediated bacterial removal attenuates staphylococcus-driven cytotoxic and pro-inflammatory effects in vitro (Fig 2B–2I).

### Bentonite prevents pyoderma development in vivo

In the BALB/c murine pyoderma model, pre-treatment of *S. pseudintermedius* D1 with 10% bentonite significantly suppressed lesion formation. The lesion area was substantially smaller in the bentonite group compared with untreated controls (p < 0.05), and quantitative bacterial counts from skin homogenates confirmed a marked reduction in staphylococcal load. Histopathological evaluation supported these findings, with bentonite-pretreated infections showing minimal epidermal hyperplasia, absence of abscess formation, and reduced inflammatory infiltration relative to controls (Table 1). Furthermore, expression of TNFα, IL-1β, and IL-13 in lesional skin was significantly downregulated in bentonite-treated groups, indicating reduced inflammatory activation (Fig 3B–3F).

**Table 1:** Histological evaluation of the back skin of a mouse model of pyoderma.

	Untreated SP	0% bentonite	10% bentonite
Epidermis			
Hyperplasia in keratinized layer	1.00 ± 0.00	1.00 ± 0.32	0.00 ± 0.00 ^*p* = 0.0022^
Hyperplasia in non-keratinized layer	2.00 ± 0.00	1.60 ± 0.24	0.00 ± 0.00 ^*p* < 0.0001^
Dermis			
Abscess	2.80 ± 0.20	2.60 ± 0.24	0.00 ± 0.0 ^*p* < 0.0001^
Inflammatory cell infiltration	1.80 ± 0.20	2.40 ± 0.40	0.20 ± 0.20 ^*p*= 0.0001^
Muscle layer			
Inflammatory cell infiltration	1.80 ± 0.37	2.60 ± 0.24	0.00 ± 0.00 ^*p* < 0.0001^
Degeneration	1.40 ± 0.40	2.00 ± 0.32	0.00 ± 0.00 ^*p*= 0.0004^
Gram-positive bacteria	2.40 ± 0.24	1.00 ± 0.00	0.00 ± 0.00 ^*p* = 0.0003^

A histological score (0, within normal limits; 1, mild; 2, moderate; 3, severe) was assigned to each observation. Results are expressed as the mean ± standard error of the mean. n = 5 per group. *p* < 0.05 (Šídák’s multiple comparisons test) vs. the 0% bentonite group.

### Topical bentonite treatment shows limited efficacy in atopic dermatitis

To assess therapeutic effects beyond bacterial sequestration, bentonite was tested in a TDI-induced model of AD. Daily topical application of a 10% bentonite gel significantly reduced ear thickness compared with vehicle-treated controls (p < 0.05). However, no improvements were observed in transepidermal water loss (TEWL) or overall clinical dermatitis scores throughout the treatment period. While bentonite displayed some anti-inflammatory benefit by reducing swelling, it did not restore barrier function or markedly alleviate AD severity (Fig 4B–4E).

## Discussion

Staphylococci are central pathogens in both human and veterinary dermatology, contributing to recurrent and treatment-resistant infections. In the present study, we provide novel evidence that sodium bentonite, a naturally occurring clay mineral with established detoxifying capacity against toxins and chemicals, directly binds staphylococci and thereby suppresses bacterial growth, cytotoxicity, and inflammation. Our findings demonstrate that bentonite pre-treatment of *S. aureus* and *S. pseudintermedius* reduced bacterial proliferation *in vitro*, alleviated keratinocyte injury and cytokine release, and prevented lesion formation in a murine pyoderma model. Importantly, these effects extended to methicillin-resistant *S. pseudintermedius* strains, highlighting the potential of bentonite as an antibiotic-independent preventive tool. However, when applied topically in a chronic AD model, bentonite showed only limited efficacy, reducing ear swelling but failing to improve TEWL or clinical severity. This dual outcome highlights both the strengths and limitations of bentonite as a candidate intervention for staphylococci-associated skin diseases.

A central observation of this work is the rapid and concentration-dependent reduction in viable *S. aureus* and *S. pseudintermedius* following brief exposure to bentonite. Importantly, our supplemental experiments confirm that this reduction does not reflect bactericidal or bacteriostatic activity: bacteria recovered from bentonite pellets remained viable and proliferated normally once separated from the clay. These results support a mechanism based on physical sequestration, consistent with the established physicochemical properties of bentonite. Bentonite-exposed bacteria resumed normal proliferation after removal of the clay, indicating sequestration rather than bacteriostasis, and SEM imaging revealed preserved bacterial morphology without evidence of cell lysis. These observations reinforce that bentonite does not exert intrinsic bactericidal or bacteriostatic activity but instead reduces recoverable CFU through adsorption and aggregation. Its negatively charged smectite platelets, high cation-exchange capacity, large specific surface area, and layered structure create optimal conditions for adsorption of bacterial surfaces enriched in cationic teichoic acids and peptidoglycan. SEM analysis in our study corroborates this mechanism by showing pronounced aggregation of bacteria around bentonite particles and reduced extracellular matrix. Although these findings strongly suggest electrostatic and adsorption-based interactions, we acknowledge that detailed surface characterization—including zeta potential measurements, adsorption isotherms, and high-resolution imaging—will be essential to fully elucidate the binding mechanism. We have added this limitation and future direction accordingly.

Pyoderma is among the most common canine skin disorders and an important challenge in veterinary practice [17]. Its recurrence is often linked to underlying conditions such as AD, endocrine disease, or ectoparasite infestations. Treatment relies heavily on antibiotics, but multidrug-resistant *S. pseudintermedius* is increasingly prevalent worldwide [18]. This resistance crisis has created demand for non-antibiotic strategies that can prevent or reduce bacterial colonization without fostering further resistance. Our murine pyoderma model demonstrated that bentonite pre-treatment of *S. pseudintermedius* markedly reduced lesion development, bacterial load, and histological markers of tissue damage. These findings demonstrate strong preventive potential and are particularly relevant given the increasing burden of methicillin-resistant *S. pseudintermedius* in veterinary dermatology. However, our model utilized bacteria pretreated with bentonite rather than topical post-infection treatment. This design was chosen because consistent colonization of canine or human staphylococci on murine skin proved difficult to establish, limiting our ability to evaluate therapeutic application. Future development of post-infection or chronic pyoderma models—potentially using species-adapted microbiota, altered barrier conditions, or repeated inoculation—will be crucial to determine whether bentonite can function therapeutically rather than solely preventively.

While bentonite showed robust preventive effects against pyoderma, its therapeutic utility in atopic dermatitis appeared limited. In the TDI-induced AD model, daily topical application of bentonite gel reduced ear thickness but did not improve TEWL or clinical severity. These findings indicate that while bentonite can modestly dampen acute inflammation, it is insufficient to resolve the complex immunological and barrier defects characteristic of AD. Several factors may explain this limited efficacy. First, AD is not solely driven by staphylococcal colonization; it involves genetic predispositions, barrier dysfunction, dysregulated immune responses, and environmental triggers. While *S. aureus* exacerbates AD, simply reducing bacterial presence may not reverse established disease. Second, bentonite acts primarily at the surface by binding extracellular bacteria. It does not penetrate skin layers or directly modulate immune pathways. In chronic AD, inflammation becomes self-sustaining, and microbial removal alone may not halt disease progression. Finally, the gel formulation used may have limited skin contact or adherence, reducing efficacy. Nevertheless, the reduction in ear thickness suggests that bentonite retains some anti-inflammatory benefit, possibly by reducing secondary bacterial burden. Its role may therefore be better suited for prevention or adjunctive therapy rather than standalone treatment in AD.

Current alternatives to antibiotics for staphylococcal skin infections primarily rely on antiseptics such as chlorhexidine, povidone-iodine, and ozone water [11,19,20]. These agents are effective at reducing bacterial burden, but their use is limited by several drawbacks. Chlorhexidine, though widely used, can be cytotoxic to mammalian cells with repeated exposure and may disrupt the skin barrier [21]. Povidone-iodine is effective but can cause skin irritation and staining, limiting compliance [22]. Ozone water, as demonstrated in recent experimental models, exhibits bactericidal activity against both *S. aureus* and methicillin-resistant *S. pseudintermedius*; however, high concentrations are required to achieve therapeutic efficacy [11]. These concentrations are difficult to generate and maintain, and ozone is inherently unstable, degrading rapidly in aqueous solutions. Moreover, exposure to high ozone levels may induce oxidative stress in host tissues, raising concerns regarding safety in chronic or preventive use. In contrast, bentonite offers several practical advantages over conventional antiseptics. First, safety: it is already approved in the European Union as a feed additive without restriction, underscoring its biocompatibility and low toxicity. Second, stability: unlike ozone, bentonite is chemically stable, easy to transport, and does not lose activity during storage. Third, its non-selective mechanism—physical adsorption of bacterial cells rather than interference with biochemical pathways—reduces the likelihood of selecting for resistant strains, an important consideration in the context of growing antimicrobial resistance. Nevertheless, bentonite is not without limitations. Unlike bactericidal agents, it does not actively kill staphylococci. Instead, its efficacy depends on sufficient binding capacity; if the bacterial load exceeds this threshold, residual organisms may persist. Furthermore, its effectiveness is influenced by concentration, formulation, and contact time, meaning heavily colonized or exudative lesions may limit its ability to neutralize pathogens. Optimizing delivery systems to prolong skin contact and maximize adsorption is therefore critical to translating bentonite’s promise into clinical utility.

Although this study focused on *Staphylococcus* species due to their central role in pyoderma and AD, our findings suggest that the sequestration effect of bentonite is not restricted to staphylococci. In preliminary assessments, we observed comparable adsorption of Gram-negative bacteria, including the periodontal pathogen *Porphyromonas gingivalis*, indicating that the physical binding mechanism may operate across a broader range of microorganisms. Nonetheless, differences in cell-surface charge distributions, outer membrane structures, and cell-wall compositions among bacterial species are likely to influence the efficiency of adsorption. Gram-positive bacteria, such as *Staphylococcus* spp., possess abundant exposed cationic domains—particularly within teichoic acids—that may enhance their interaction with the negatively charged surfaces of bentonite. These variations may explain the particularly robust sequestration observed in staphylococci in this study. Future work will systematically compare bentonite–microbe interactions across diverse taxa to define the breadth and limits of this physical binding mechanism.

The findings of this study point to several potential clinical applications for sodium bentonite. As a preventive cleansing agent, it could be incorporated into shampoos, sprays, or wipes for dogs predisposed to recurrent pyoderma, reducing bacterial load before infection develops. As an adjunctive therapy, bentonite may be combined with antibiotics or anti-inflammatory drugs to enhance bacterial clearance while reducing drug use and resistance risk. Its strong adsorptive properties also support use as a barrier formulation in wound dressings or surgical coatings to limit bacterial adhesion and protect healing tissues [23]. Importantly, regular application in companion animals may contribute to zoonotic control, lowering the risk of methicillin-resistant *S. pseudintermedius* (MRSP) or *S. aureus* transmission to humans [24]. Nevertheless, effective clinical use will require species-specific formulations that account for differences in skin thickness, lipid composition, follicular density, and pH between mice, humans, and dogs [25,26]. Vehicle type, hydration state, and contact time are critical for maintaining bacterial adsorption, and murine models may not fully replicate chronic disease in canine patients.

The findings of this study suggest that sodium bentonite sequesters *Staphylococcus* species primarily through a physical, nonspecific adsorption mechanism. Bentonite’s high specific surface area, negatively charged platelet surfaces, and strong cation-exchange capacity create an environment conducive to electrostatic attraction and van der Waals interactions with bacterial cell surfaces. *Staphylococcus* species, including *S. pseudintermedius* and *S. aureus*, possess abundant positively charged components within their thick peptidoglycan layers and teichoic acids, which likely enhance their affinity for the negatively charged smectite surfaces. In addition to these electrostatic interactions, the layered structure of bentonite may facilitate mechanical entrapment of bacterial cells within microaggregates, further strengthening sequestration. Consistent with this interpretation, our SEM observations demonstrated bacterial clustering around bentonite particles and a reduction in extracellular matrix after exposure. Although these findings support a physical binding mechanism, we acknowledge that detailed physicochemical characterization—such as surface charge measurements, adsorption isotherms, and high-resolution imaging—will be essential to fully elucidate the underlying interactions.

While this work provides novel insights into the preventive potential of sodium bentonite against staphylococcal infections, several limitations should be acknowledged. First, the strong binding and inhibitory effects observed *in vitro* may not fully translate to the complex skin environment, where factors such as sebum, hair, and exudates could reduce efficacy. Second, the study assessed only short pre-treatment times, whereas real-world application may require longer or repeated exposures to achieve comparable outcomes. Third, the formulation used in the AD model—a 10% bentonite gel—may not represent the most effective delivery system; alternative vehicles such as sprays, shampoos, or pastes might improve skin adherence and therapeutic performance. Fourth, our experimental models focused on acute pyoderma and chemically induced AD, which do not fully capture the complexity of recurrent or mixed infections seen clinically. Fifth, the impact of bentonite on the commensal skin microbiota was not assessed, an important consideration for maintaining long-term skin health.

Finally, although the proposed adsorption-based mechanism aligns with the well-established physicochemical properties of bentonite—including its large specific surface area, layered platelet structure, and high cation-exchange capacity [27,28]—we did not directly characterize changes in these properties following bacterial binding. Measurements such as BET surface area analysis, pore size distribution, and dynamic light scattering to assess particle size and aggregation would provide quantitative insight into the extent of bacterial adsorption and structural alterations of the clay. Prior studies indicate that both particle size and the degree of smectite exfoliation can influence microbial adhesion by modulating accessible surface area and charge interactions. We therefore recognize the absence of such measurements as a limitation and identify this as an important direction for future research, where systematic characterization of bentonite’s physical properties during bacterial interaction will help clarify the underlying mechanisms.

Future research should focus on optimizing bentonite for therapeutic use by developing advanced formulations such as shampoos, hydrogels, or nanoparticle-enhanced clays to improve skin adherence and bacterial binding. Comprehensive dose–response and safety studies in animals and humans are needed to assess long-term irritation, systemic absorption, and effects on the skin microbiome. Investigating bentonite in combination with antibiotics, antiseptics, or barrier-enhancing agents may reveal synergistic effects, particularly in atopic dermatitis, while mechanistic studies could clarify its interactions with staphylococcal surface proteins and toxins. Ultimately, well-designed clinical trials in dogs with pyoderma and humans with recurrent staphylococcal infections are essential to establish real-world efficacy and guide clinical application.

## Conclusion

This study identifies sodium bentonite as a promising non-antibiotic strategy for preventing staphylococci-associated skin diseases. By directly binding bacteria, bentonite reduces growth, cytotoxicity, and inflammation in vitro and prevents pyoderma in vivo. Its safety, stability, and antibiotic-independent mechanism position it as an attractive preventive option, particularly in veterinary dermatology where recurrent pyoderma and antibiotic resistance are major concerns. However, its limited efficacy in chronic AD underscores the need for targeted application, optimized formulations, and integration into broader therapeutic strategies. Future translational studies will determine whether bentonite can bridge the gap between experimental promise and clinical utility in both humans and companion animals.

## Supporting information

S1 File Raw data for Figs 1–4. (XLSX)
